# A Comprehensive Assessment of Renal Function During and After Pulsed Field Ablation

**DOI:** 10.1111/jce.70281

**Published:** 2026-04-14

**Authors:** Václav Melenovský, Marek Hozman, Sabri Hassouna, Ivana Fišerová, Barbora Bačová, Dalibor Heřman, Jakub Karch, Jana Veselá, Michal Zuzčák, Ivan Rychlík, Pavel Osmančík

**Affiliations:** ^1^ Department of Cardiology University Hospital Kralovske Vinohrady, 3rd Faculty of Medicine, Charles University Prague Czech Republic; ^2^ Department of Biochemistry and Molecular Biology, 3rd Faculty of Medicine Charles University Prague Czech Republic; ^3^ Department of Laboratory Hematology, Central Laboratories, 3rd Faculty of Medicine University Hospital Kralovske Vinohrady Prague Czech Republic; ^4^ Departement of Internal Medicine, University Hospital Kralovske Vinohrady, 3rd Faculty of Medicine Charles University Prague Czech Republic

**Keywords:** acute kidney injury, atrial fibrillation, catheter ablation, heme, pulsed‐field ablation

## Abstract

**Background:**

Acute kidney injury is an uncommon complication of pulsed field ablation (PFA). There is, however, limited empirical data on the dynamics of renal biomarkers apart from creatinine. Furthermore, no study to date has assessed creatinine at more than 24 h after the procedure.

**Methods:**

Patients undergoing PFA for atrial fibrillation (AF) were enrolled in a prospective observational study. The ablation was performed using the *Farawave* pentaspline catheter along with the *Farapulse* system and consisted of pulmonary vein isolation along with additive lesions in patients with persistent AF and/or atrial flutter. Administration of 500–1000 mL of crystalloid was a mandatory part of the periprocedural management protocol. Before and 24 h after the procedure, serum renal biomarkers were measured (urea, creatinine, cystatin C, NGAL, and KIM‐1), and urinalysis was performed. Hemolysis indices (haptoglobin, Lactate Dehydrogenase, and red blood cell microparticles) were measured after the procedure. Patients were instructed to undergo blood sampling for creatinine and urea 48 h after the procedure.

**Results:**

Samples from 88 patients were analyzed (mean age 67 ± 8.8 years, 33% female, 53% paroxysmal AF, average of 63 [IQR: 40–77] applied pulses). The creatinine concentration at 24 h decreased from 86 (IQR: 72–100) to 82 (IQR: 68–92) µmol/L (*p *< 0.001), the concentration at 48 h was similar to preprocedural values (83 [IQR: 69–100] µmol/L; (*p *= 1.00). However, 9 patients (10.2%) met AKI criteria; in six of them, AKI was only detectable at the 48‐h measurement. Serum cystatin‐C levels remained unchanged, and urea and urinary erythrocytes increase significantly from baseline to 24 h. NGAL levels increased significantly from 121 (IQR: 86–173) to 143 (IQR: 112–198) at 24 h while changes in KIM‐1 were unsignificant. All changes in renal function were transient and did not require hospitalization. Logistic regression identified age and the total number of pulsed field pulses as the major risk factors for AKI.

**Conclusions:**

Routine PFA with periprocedural hydration and an average of 63 PF pulses appears safe in patients without significant preexisting renal disease, with a low risk of clinically relevant renal impairment. However, the incidence of AKI in our cohort was higher than previously reported, suggesting that transient kidney function changes may be more common than recognized. These findings highlight the need for kidney function monitoring beyond 24 h in higher‐risk patients undergoing PFA.

**Abbreviations**
AF: atrial fibrillation
AKI: acute kidney injury
CKD: chronic kidney disease
KDIGO: kidney kisease: improving global outcomes organization
KIM‐1: kidney injury molecule 1
NGAL: neutrophil gelatinase‐associated lipocalin
PFA: pulsed field ablation

## 1 Introduction

In contrast to thermal energy catheter ablation (cryoballoon or radiofrequency), pulsed field ablation (PFA) leads to cardiomyocyte death due to cell membrane electroporation [1]. PFA procedures are faster, and since PF energy is associated with less extracardiac tissue damage (such as atrio‐esophageal fistula), it is becoming increasingly more utilized in catheter ablation of cardiac arrhythmias [2]. Trials and registers document the efficacy and safety of PFA [2, 3]. However, real‐life experience and registry data have shown other adverse events which were not present with thermal energy. One of these is heme‐mediated acute kidney injury (AKI). AKI after PFA was first described in late 2024, and data on the exact incidence of AKI and its temporal association to the procedure are relatively scarce. In an observational study of over 17 000 patients undergoing PFA, the incidence of heme‐related renal failure necessitating transient hemodialysis treatment occurred in 0.03% patients [2]. However, other recent observational studies reported an overall AKI incidence between 1% and 3% [4, 5]. Since the original observations of the association of PFA with heme‐induced AKI, multiple groups, including ours, have published on the direct association of PFA and hemolysis and subsequent changes in renal function [4, 5, 6, 7, 8].

So far, published studies focused only on 1‐day change in renal function, and have not reported on renal function after the 24 h time horizon after PF ablation. Importantly, studies on contrast‐induced nephropathy have shown that relying solely on 24‐h measurements can miss up to 58% of cases that would otherwise be detected at 48 h postprocedure [9, 10]. Our hypothesis was that most AKI following PFA would also manifest more than 24 h after the intervention. Thus, the aim of the present study was to perform comprehensive assessment of renal function after PFA and to expand the examinations beyond the 24‐h time frame.

## 2 Methods

This was a prospective observational single‐center study. The study protocol was approved by the institutional ethical review board of University Hospital Kralovske Vinohrady in accordance with The Declaration of Helsinki. Eligible patients were patients undergoing planned PFA for atrial fibrillation (AF) according to current guidelines. The only inclusion criterion was an indication for catheter ablation for AF, signed informed consent, and age over 18 years. Exclusion criteria were history of hematological disease (excluding iron deficiency anemia) and chronic kidney disease stage IV or V. Preprocedural chronic kidney disease chronic kidney disease (CKD) I–III was not an exclusion criterion.

AKI was defined according to the 2011 kidney disease: Improving global outcomes (KDIGO) Clinical Practice Guideline for AKI [11]. KDIGO defines AKI as either an absolute increase in serum creatinine by at least 26.5 µmol/L (i.e., ≥ 0.3 mg/dL) within 48 h or a relative increase of serum creatinine by at least 1.5 times baseline within the prior 7 days or a decrease in urine volume below 0.5 mL/kg/h for 6 h. Our study did not utilize the final condition as we did not assess urine output. KDIGO AKI definitions and AKI stages can be seen in Table 1.

**Table 1:** AKI definitions and stage.

KDIGO AKI definition
Increase in serum creatinine by ≥ 26.5 µmol/L (i.e., ≥ 0.3 mg/dL) within 48 h OR
Increase of serum creatinine by ≥ 1.5x baseline within the prior 7 days

KDIGO AKI Stages	
**Stage 1:**	1.5–1.9x baseline OR ≥ 26.5 µmol/L (i.e., ≥ 0.3 mg/dL) increase
**Stage 2:**	2.0–2.9x baseline
**Stage 3:**	3.0x baseline OR ≥ 353.6 mmol/L (≥ 4.0 mg/dL) increase OR initiation of renal replacement therapy

Abbreviations: AKI, acute kidney injury; KDIGO, kidney disease: Improving global outcomes organization.

### 2.1 The Ablation Procedure

Procedures were performed under general anesthesia or deep analgosedation under the discretion of the anesthesiologist. Two or three femoral venous accesses (F16, F11, F7) were achieved under ultrasonographic guidance. Transseptal access was achieved with the help of intracardiac echocardiography using an SL1 sheath (Abbott, USA), which was subsequently replaced by the dedicated ablation sheath (Faradrive, Boston Scientific) using the over‐the‐wire technique. A bolus of 5000 IU of heparin was administrated before transeptal puncture, additional heparin bolus was given after transseptal puncture according to body weight to achieve an activated clotting time > 300 s during ablation. Pulsed‐field ablation was performed using a pentaspline catheter (Farawave, Boston Scientific) and a PFA generator (Farastar, Boston Scientific). In paroxysmal AF patients, only pulmonary vein isolation (PVI) was performed, which consisted of four PF applications in the basket and four applications in the flower configuration for each vein. PVs were checked for the entrance block; if the block was not present, additional PF applications were given. In nonparoxysmal AF, posterior wall isolation and posterior mitral isthmus ablation were performed along with PVI. Posterior wall isolation consisted of two rows of three pairs of overlapping lesions between the left and right‐sided veins, mitral isthmus isolation was performed between the left inferior PV and mitral anulus by 9–20 PF application. Electrical cardioversion was performed at the end of the procedure in patients in AF.

Patients with documented typical atrial flutter also underwent cavotricuspid isthmus ablation with PFA. The exact number of applications was recorded in all patients. All catheters and sheaths were removed at the end of the procedure, and the access sites were fixed using *Z‐*stitches.

Since sufficient hydration is crucial to prevent worsening of renal function post‐procedure, great attention was paid to adequate hydration. As per protocol, all patients received additional 500–1000 mL of 0.9% NaCl intravenously after the procedure and were instructed about the importance of adequate oral fluid intake in the afternoon/evening after the procedure. Patients were discharged 1 day after the procedure.

### 2.2 Urinalysis

On the day of the procedure (baseline) and 1 day after the procedure (24 h), morning urine samples were obtained for urinalysis. Urinary albumin concentration was determined using the commercial immunoturbidimetry Atellica CH Microalbumin_2 assay. Urine concentrations of total protein and creatinine were measured using commercial absorption spectrophotometry assays (Atellica CH Urinary/Cerebrospinal Fluid Protein and Atellica CH Creatinine_2). All analytes were measured using the Atellica Solution Analyzer (Siemens Healthineers).

Chemical and morphological analysis of urine samples was performed using the Sysmex UN‐Series urinalysis workflow (Sysmex Corporation). According to institutional standards, the urinalysis is verified by optical microscopy using the Sternheimer stain when instrument findings are inconclusive and not all particles can be accurately classified into specific groups by the analyzer.

### 2.3 Blood Sampling

Venous blood samples were obtained at baseline before the onset of anesthesia (baseline), immediately postprocedure before sheath removal (end‐of‐procedure), and in the morning 1 day after the procedure (hereafter referred to as 24 h). The last blood sample was performed on an out‐patient basis 1–2 days after discharge (hereafter 48 h; specifically Friday for procedures performed on Wednesday and Monday for procedures performed on Friday). Renal function biomarkers (serum creatinine, urea, and cystatin C) were analyzed using the Atellica Solution Analyzer (Siemens Healthineers) and were assessed at baseline, 24 h, and 48 h (without cystatin C at 48 h). Plasma concentrations of neutrophil gelatinase‐associated lipocalin (NGAL) and kidney injury molecule 1 (KIM‐1) were determined at baseline, end‐of‐procedure, and 24 h samples using enzyme‐linked immunosorbent assays (ELISA; R&D Systems and ElabScience, USA). ELISAs were performed according to the manufacturer's protocols.

At the end of the procedure, the extent of hemolysis was estimated by measuring red blood cell microparticles (RBCµ), lactate dehydrogenase (Atellica Solution Analyzer, Siemens Healthineers), and haptoglobin (Atellica NEPH 360 System, Siemens Heathineers). The concentration of RBCµ, fragments of damaged erythrocytes, was assessed from samples of citrated whole blood using flow cytometry (identified as CD235a and annexin V positive events). Details of the flow cytometry method used for RBCμ determination have been described previously [6].

### 2.4 Statistical Analysis

Baseline characteristics are presented using descriptive statistics. Continuous variables were assessed for normality (Shapiro–Wilk test) and are shown as mean ± standard deviation (SD) if normally distributed or as median and interquartile ranges (IQR) if the distribution is non‐Gaussian. Categorical variables are reported as counts and percentages. Changes in non‐normally distributed laboratory values across three time points were first assessed using the Friedman test, followed by pairwise Wilcoxon signed‐rank tests with Bonferroni adjustment for multiple comparisons (urea and creatinine at baseline, 24 and 48 h and KIM and NGAL at baseline, end of procedure and 24 h). For comparisons of continuous variables between AKI versus non‐AKI patients, the Wilcoxon rank‐sum test was used. Univariate and multivariate logistic regression was performed with AKI as a binary outcome variable. All analyses were performed using Stata version 18.5 (StataCorp LLC, College Station, TX, USA). A significance level of *p* < 0.05 was used for hypothesis testing where applicable.

## 3 Results

### 3.1 Baseline Characteristics

Between February and December of 2024, 88 patients were enrolled (29 [33%] women, age 65 ± 8.8 years, 47 [53%] with paroxysmal AF). The median number of PF pulses was 63 (IQR: 40–77). Follow‐up blood samples at 48 h were available for 55 patients (63% of the cohort). The baseline clinical characteristics are shown in Table 2.

**Table 2:** Baseline characteristics (*n* = 88).

Age (years)	67 ± 8.8
Female	29 (33%)
Paroxysmal AF	47 (53%)
Non‐paroxysmal AF	41 (47%)
eGFR—CKD‐EPI 2012 (mL/min/1.73 m^2^)*	
> 90	20 (22.7%)
60–89	53 (60.2%)
45–59	11 (12.5%)
30–44	4 (4.55%)
BMI (kg/m^2^)	30 ± 5.2
Left ventricular ejection fraction (%)	60 (IQR: 55–60)
Diabetes	19 (22%)
Arterial hypertension	60 (68%)
Heart failure	14 (16%)
History of stroke/TIA	3 (3%)
Thyroid disease	16 (18%)
Antiarrhythmic drugs	
Amiodarone	18 (20%)
Propafenone	21 (24%)
Total number of pulsed‐field pulses	63 (IQR: 40–77)
Length of ablation procedure (minutes)	60 (IQR: 47–72)
Sinus rhythm restoration during procedure	37 (42%)

Abbreviations: AF, atrial fibrillation; BMI, body mass index; TIA, transient ischemic attack.

* eGFR was calculated with creatinine concentration at baseline.

### 3.2 Creatinine, Urea, Cystatin C

**Figure 1:**
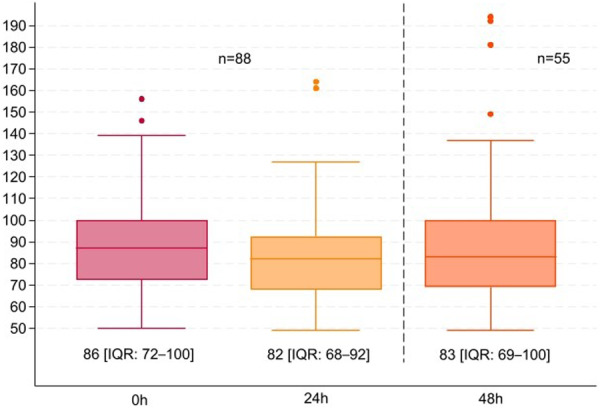
Creatinine concentrations.

The time course of differences in creatinine and urea concentrations was significant (*p* < 0.01). Compared to baseline creatinine concentration (86 [IQR: 72–100] µmol/L), a significant decrease was seen at 24 h (82 [IQR: 68–92] µmol/L; *p* < 0.001). The concentration of serum creatinine in patients (*n* = 55 [63%]) who complied with 48 h blood sampling was 83 (IQR: 69–100) µmol/L, and the difference between baseline and 48 h samples was not significant (*p* = 1.00). Figure 1 shows the comparison of creatinine concentrations. Although the difference between baseline and 24 h urea concentrations was significant (*p* = 0.002), the difference between baseline and 48 h samples did not reach statistical significance (*p* = 0.54). Cystatin C concentrations were similar at baseline and 24 h samples. The values of the most relevant renal biomarkers are seen in Table 3.

**Table 3:** Changes in creatinine, urea, and cystatin C.

	Baseline (*n* = 88/84/74)*	24 h (*n* = 88/84/74)*	48 h (*n* = 55/50/‐)*	*p* value (Baseline vs. 24 h)	*p* value (Baseline vs. 48 h)	*p* value (24 h vs. 48 h)	
Creatinine (µmol/L)	86 (72–100)	82 (68–92)	83 (69–100)	< 0.001	1.00	0.008
Urea (mmol/L)	5.65 (4.70–6.70)	6.30 (5.10–7.25)	5.70 (4.80–7.10)	0.002	0.54		
Cystatin C (mg/L)	0.97 (0.87–1.15)	0.97 (0.84–1.11)		0.40			

* Sample sizes for creatinine, urea, and cystatin C, respectively

### 3.3 AKI

Although no significant increase in mean creatinine concentrations between baseline and 24 h, or 48 h values was found, 9 in 88 patients (10.2%) postprocedurally met AKI criteria. Of these nine patients, AKI criteria were met in the 24 h sample in only three of them (33.3%), and in the 48 h sample in six (66.7%) of them. All AKI patients fulfilled the criterion of an absolute increase in serum creatinine of at least 0.3 mg/dL (26.5 mmol/L), and three of these patients also fulfilled the criterion of a relative increase in serum creatinine of 1.5 times above baseline. The trajectory of AKI patients and AKI criteria fulfillment is shown in Table 2. Applying CKD staging per eGFR, 5/9 AKI patients would be in the CKD 1 or 2 categories. All patients with AKI were followed beyond 48 h, and all changes in renal functions were transient, and the creatinine values returned to baseline values within days after PFA without the need for hospitalization. Applying the KDIGO staging criteria, all of the observed acute kidney injuries were classified as AKI stage 1. The trajectory of the AKI patients is shown in Table 4.

**Table 4:** The trajectory of AKI patients and AKI criteria fulfillment.

AKI patient No.	Baseline CKD category	Baseline creatinine (µmol/L)	24 h creatinine (µmol/L)	48 h creatinine (µmol/L)	Number of PF applications	When AKI was recognized	Type of AKI definition fulfilled	Maximum relative increase	Maximum absolute change (µmol/L)	AKI stage
1	1	52	68	79*	64	48 h only	Absolute + Relative	1.52×	27	1
2	2	100	92	194	41	48 h only	Absolute + Relative	1.94×	94	1
3	2	105	108	137	70	48 h only	Absolute only	1.31×	32	1
4	3a	116	109	192	77	48 h only	Absolute + Relative	1.66×	76	1
5	3a	112	164	127	98	24 h only	Absolute only	1.46×	52	1
6	2	112	105	149	69	48 h only	Absolute only	1.33×	37	1
7	3a	146	127	181*	49	48 h only	Absolute only	1.24×	35	1
8	3a	133	161	x	113	24 h only	Absolute only	1.21×	28	1
9	1	69	100	x	104	24 h only	Absolute only	1.45×	31	1

* Creatinine measurement performed at 72 h postprocedure.

### 3.4 Urinalysis

The results of urinalysis are shown in Table 5; complete samples were available in 77 patients. There was a significant increase in urinary erythrocytes from baseline to 24 h. The difference in urinary leukocytes was not significant. Urinary pH between baseline and 24 h was also not significantly different. Urine specific gravity increased significantly from baseline to 24 h. Similarly, albumin‐to‐creatinine ratio (ACR) and protein‐to‐creatinine ratio (PCR) both increased significantly from baseline to 24 h.

**Table 5:** Urinalysis parameters.

	Baseline	24 h	*p* value
Urinary erythrocytes (/µL)	4 (2–7)	11 (4–48)	< 0.001
Urinary leukocytes (/µL)	4 (2–19)	10 (2–51)	0.243
Urinary pH	5.5 (5.5–6)	5.5 (5.5–6)	0.852
Urine specific gravity (kg/m^3^)	1016 (1013–1021)	1022 (1016–1028)	< 0.001
ACR (mg/mmol)	0 (0–1)	0.9 (0–2.2)	0.002
PCR (mg/mmol)	10 (0–14)	12 (7–17)	0.005

Abbreviations: ACR, albumin‐to‐creatinine ratio; PCR, protein‐to‐creatinine ratio.

### 3.5 KIM and NGAL

**Figure 2:**
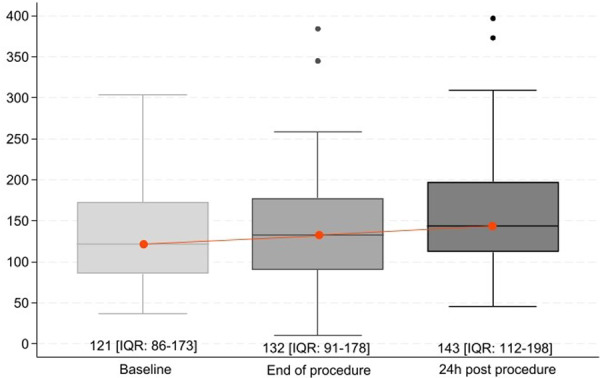
Changes in NGAL concentrations.

Measurements of NGAL and KIM were performed in 55 and 43 patients, respectively. NGAL concentrations showed a significant difference across timepoints (Friedman test, *p* < 0.01) and compared to baseline values, concentrations of NGAL increased significantly at 24 h (Figure 2 and Table 6). The Friedman test showed a significant difference across the three time points for KIM‐1 (*p* < 0.001). However, post hoc pairwise comparisons did not show significant changes of end‐of‐procedure or 24 h concentrations compared to baseline.

**Table 6:** NGAL and KIM‐1.

	*n*	Baseline	Postprocedure	24 h	*p*‐value (baseline vs. postprocedure)	*p*‐value (baseline vs. 24 h)
NGAL (ng/mL)	55	121 (86–173)	132 (91–178)	143 (112–198)	0.216	0.002
KIM‐1 (ng/mL)	43	8.6 (4.4–29)	9.1 (5.1–31.50	11.6 (4.2–26.7)	0.314	0.647

### 3.6 Hemolysis

Markers of hemolysis were measured at the end of the procedure. Median serum Lactate Dehydrogenase (LDH) levels were 3.70 (IQR: 3.25–4.44) µkat/L, haptoglobin was 0.80 (IQR: 0.64–1.09) g/L, and the concentration of RBCµ was 1585.6 (IQR: 1428.9–1942.0) RBCµ/µL.

### 3.7 Logistic Regression

To identify predictors of AKI occurrence, univariate logistic regression using selected laboratory, procedural, and clinical variables was performed. Clinical and procedural characteristics that met statistical significance (*p* < 0.05) were older age (OR 1.23; CI: 1.05–1.45; *p* = 0.010) and the number of pulsed‐field energy applications (OR 1.04; CI: 1.00–1.08; *p* = 0.030). From the baseline laboratory variables eGFR using creatinine and cystatin C were significant predictors of AKI (OR 0.05; CI 0.00–0.68; *p* = 0.025 and OR 0.02; CI: 0.00–0.44; *p* = 0.013, respectively). Considering hemolytic parameters, LDH at the end of procedure was also a significant predictor of AKI OR 2.01; CI: 1.05–3.82; *p* = 0.034). Avoiding overfitting given the limited number of endpoints, a final multivariate model of age, baseline creatinine eGFR, and PF applications was created. The adjusted OR for age was 1.22 (1.00–1.48; *p* = 0.040) and 1.05 (1.01–1.1; *p* = 0.027) for the number of PF applications; baseline eGFR did not retain significance. A sensitivity analysis of the predictive value of the number of PF applications was performed. In the receiver operating characteristic (ROC) analysis, the area under the curve (AUC) is 0.706, showing a moderate predictive value. The highest sensitivity versus specificity was observed at around 70 pulse field energy applications, which is consistent with the cutoff for significant hemolysis proposed by Venier et al. [8].

## 4 Discussion

The main findings of our analysis is that a significant increase of serum creatinine meeting AKI criteria after pulsed‐field ablation of AF using a pentaspline catheter with a median number of ~63 PF applications is rare and is present in mild form in 10% of patients. Importantly, but not surprisingly, AKI is more often present beyond the timeframe of 24 h. Of particular relevance is that using adequate hydration, concentrations of creatinine at 24 h after the procedure are even lower compared to baseline values, which may lead to an underestimation of renal function monitoring.

### 4.1 The Temporal Relationship Between AKI and PFA

Our work builds on our previous work on PFA‐associated hemolysis [6, 12]. As opposed to other studies, our work explored the relationship between PFA and AKI beyond the usual 24 h horizon and included an exploratory analysis of different kidney injury biomarkers. A noteworthy finding is that overall, creatinine concentration at 24 h decreased statistically significantly. This can be almost fully explained by the periprocedural parenteral hydration (in conjunction with unrestricted oral fluid intake). Sinus rhythm restoration in AF patients is known to improve renal function [13]. However, such improvements are observed in the long term, the observed decrease in serum creatinine after the procedure is very unlikely to be caused by sinus rhythm restoration. Moreover, most patients were in sinus rhythm during the procedure, and cardioversion from AF to SR was present only in 42% of patients. Since peri‐procedural hydration became a standard preventive measure during PF ablation, the falsely reassuring improvement in renal function at 24 h can have clinical consequences, as it could lead to the underestimation of renal injury in high‐risk patients who received peri‐procedural hydration, and clinicians dealing with PF ablations should be aware of it.

Although the pathophysiological mechanism differs, kidney damage caused by hemolysis has several similarities with contrast‐induced nephropathy. Both entities usually develop more than 24 h after the initial noxious stimulus. Analogous to contrast‐induced nephropathy, AKI after PFA is also dose‐dependent—a higher number of PF pulses leads to a greater extent of hemolysis and presents a higher risk for AKI [9].

The incidence of AKI in our cohort was higher than previously reported, suggesting that transient kidney function changes beyond the horizon of 24 h may be more common than recognized to date. AKI was present in 10% of the patients in our cohort. It is, however, important to emphasize that most patients had only a mild decline in eGFR classifying as stage I AKI using the KDIGO definition. Moreover, outcomes were good in all patients, and serum creatinine returned to baseline levels in days or weeks after the procedure. The long‐term impact of mild and temporary worsening in postprocedural renal function is unclear. On one hand (as observed with contrast‐induced nephropathy), transient kidney injury is known to be a risk factor for later CKD development [14]. On the other hand, in the long term, the benefits of sinus rhythm restoration could outweigh these increased risks.

### 4.2 Mechanism of AKI After PFA

Contemporary understanding of heme pigment‐associated kidney injury postulates that direct tubular cell injury, tubular obstruction, and hemodynamic changes leading to ischemic injury are the main pathophysiological culprits. Mounting evidence suggests that the elevated intravascular heme pigment concentration is the main culprit in AKI after PFA [2, 4, 5, 7, 8]. In the present study, hemolysis markers including RBC microparticles were determined, which represent a highly specific and sensitive marker of hemolysis. The associated increase in LDH, urinary erythrocytes, and the decrease in haptoglobin observed after the procedure are complementary markers of acute hemolysis and are consistent with previous reports. In animal models of heme‐induced kidney injury, heme has been shown to directly reduce renal cortical blood flow [15]. This corresponds to the increase in urinary erythrocyte concentration at 24 h, which is likewise in agreement with prior findings. This may explain our observation that despite sufficient hydration, plasmatic urea concentrations significantly increased at 24 h versus baseline, which would be expected in a transient renal hypoperfusion state where passive reabsorption of urea follows the increased reabsorption of sodium and water in the proximal tubule. The majority of patients experienced an increase in NGAL and KIM1 levels, and a trend towards an increase was seen immediately after the procedure, further supporting the concept of prompt periprocedural tubular stress. However, only the change of NGAL from baseline to 24 h postprocedure reached statistical significance. Due to sampling and analysis limitations, these biomarkers were assessed only in a small subset, which likely explains the lack of significance. NGAL has proven to be an early and accurate biomarker of renal tubular injury and AKI. Several reports have indicated that plasma NGAL has superior predictive value as compared to urine NGAL [16, 17]. Urinary and plasma KIM‐1 also have established utility in early AKI prediction [18, 19]. A recognized risk factor for heme‐induced kidney injury is low urinary pH, which facilitates heme precipitation and subsequent cast formation [20]. However, in our cohort, baseline pH in AKI patients was similar compared to non‐AKI patients. Another established AKI risk factor is dehydration. Likewise, urine specific gravity at baseline (serving as a surrogate for hydrations status) was not different between AKI and non‐AKI patients. It is to note that all patients with preexisting renal disease were excluded, and all patients were admitted on an elective basis in a standard hydration status. Increased albuminuria and proteinuria in the absence of established CKD can serve as markers of subclinical kidney disease [21]. In univariate regression, neither ACR or PCR at baseline were predictive of AKI. The observed increase in ACR and PCR after the procedure can be partially explained by analytical interference from heme‐related compounds. Albuminuria has, however, been recognized as a possible biomarker in AKI [22].

### 4.3 Who Is at Higher Risk of AKI After PFA?

Based on univariate regression, age, baseline eGFR, end‐of‐procedure LDH, and the number of PF applications were statistically significant predictors of AKI. In a multivariate model, PF applications and age were the only to retain significance. Sensitivity analysis confirmed the predictive role of the number of PF applications. We propose 70 applications as a possible cutoff for higher AKI risk. Nevertheless, AKI was likewise observed in patients who received a much lower amount of applications and AKI risk must be viewed as a composite of patient‐level and procedural risk factors. In previous works, the extent of hemolysis was proportional to the amount of PF applications. Along with the predictive role of LDH, this further underlies the dose‐dependent effect of PF energy‐induced hemolysis on AKI risk, and important role of age as a risk factor for AKI. The critical role of adequate periprocedural hydration including avoiding unnecessarily prolonged nil‐per‐os and parenteral fluid administration during and shortly after the procedure must once again be stressed. Evidence for the renoprotective effect of hydration comes from decades of work on treatment of rhabdomyolysis patients or from observational studies in PFA [7, 23].

## 5 Limitations

The major limitation of this study is the small sample size. Not all patients were able to undergo outpatient blood sampling after 24 h. Data at 48 h postprocedure required patients to commute to a blood draw center near their home, and this was inconvenient for many patients. Another limitation is the lack of precise monitoring of fluid intake and diuresis. Importantly, patients with CKD 3 or 4 were not included, and our results cannot be representative of the patient group with a higher degree of preprocedural renal kidney dysfunction. Although these patients were not included, the delayed AKI occurrence is an important findings, and exactly high‐risk renal patients (i.e., CKD4‐5) should be monitored more carefully, beyond the 24 h period. Finally, the current data is limited to the FARAPULSE™ system and cannot be directly extrapolated to other PFA systems, as evidence suggests that the extent of hemolysis and other periprocedural characteristics differ between the PFA modalities [24, 25].

## 6 Conclusion

PFA in our cohort without significant baseline renal disease, standard periprocedural hydration, and an average of 63 pulsed field energy applications proved safe. However, the incidence of AKI in our cohort was higher than previously reported, suggesting that transient kidney function changes may be more common than recognized to date. The number of PF energy applications and age are risk factors for AKI. Measures to prevent periprocedural dehydration and follow‐up testing of kidney function, especially in higher risk individuals, is warranted beyond 24 h.

## Ethics Statement

Approved by the institutional ethical review board of University Hospital Kralovske Vinohrady.

## Consent

All participants provided written informed consent before inclusion in the study.

## Conflicts of Interest

The authors declare no conflicts of interest.

## References

[1] A. Sugrue , E. Maor , A. Ivorra , et al., “Irreversible Electroporation for the Treatment of Cardiac Arrhythmias,” Expert Review of Cardiovascular Therapy 16, no. 5 (2018): 349–360, 10.1080/14779072.2018.1459185. 29595355

[2] E. Ekanem , P. Neuzil , T. Reichlin , et al., “Safety of Pulsed Field Ablation in More Than 17,000 Patients With Atrial Fibrillation in the MANIFEST‐17K Study,” Nature Medicine 30, no. 7 (2024): 2020–2029, 10.1038/s41591-024-03114-3. PMC11271404 38977913

[3] V. Y. Reddy , E. P. Gerstenfeld , A. Natale , et al., “Pulsed Field or Conventional Thermal Ablation for Paroxysmal Atrial Fibrillation,” New England Journal of Medicine 389, no. 18 (2023): 1660–1671, 10.1056/NEJMOA2307291/SUPPL_FILE/NEJMOA2307291_DATA-SHARING.PDF. 37634148

[4] M. A. Popa , S. Venier , R. Menè , et al., “Characterization and Clinical Significance of Hemolysis After Pulsed Field Ablation for Atrial Fibrillation: Results of a Multicenter Analysis,” Circulation. Arrhythmia and Electrophysiology 17, no. 10 (October 2024): e012732, 10.1161/CIRCEP.124.012732. 39212069

[5] F. Jordan , S. Knecht , C. Isenegger , et al., “Acute Kidney Injury After Catheter Ablation of Atrial Fibrillation: Comparison Between Different Energy Sources,” Heart Rhythm 21, no. 8 (2024): 1248–1249, 10.1016/j.hrthm.2024.04.046. 38608916

[6] P. Osmancik , B. Bacova , D. Herman , et al., “Periprocedural Intravascular Hemolysis During Atrial Fibrillation Ablation,” JACC: Clinical Electrophysiology 10, no. 7 (2024): 1660–1671, 10.1016/J.JACEP.2024.05.001. 38852101

[7] S. Mohanty , M. Casella , P. Compagnucci , et al., “Acute Kidney Injury Resulting From Hemoglobinuria After Pulsed‐Field Ablation in Atrial Fibrillation,” JACC: Clinical Electrophysiology 10, no. 4 (2024): 709–715, 10.1016/J.JACEP.2023.12.008. 38310489

[8] S. Venier , N. Vaxelaire , P. Jacon , et al., “Severe Acute Kidney Injury Related to Haemolysis After Pulsed Field Ablation for Atrial Fibrillation,” Europace 26, no. 1 (2023): euad371, 10.1093/EUROPACE/EUAD371. 38175788 PMC10776308

[9] D. Reddan , M. Laville , V. D. Garovic , D. Reddan , and M. Laville , "Contrast‐Induced Nephropathy Contrast‐Induced Nephropathy and Its Prevention: Its Prevention: What Do We Really Know From Evidence‐Based Findings? Do We Really Know From Evidence‐Based Findings?," 2025, https://www.lenus.ie/handle/10147/94204. 19557710

[10] C. J. Davidson , M. Hlatky , K. G. Morris , et al., “Cardiovascular and Renal Toxicity of a Nonionic Radiographic Contrast Agent After Cardiac Catheterization. A Prospective Trial,” Annals of Internal Medicine 110, no. 2 (1989): 119–124, 10.7326/0003-4819-110-2-119. 2909204

[11] J. A. Kellum , N. Lameire , P. Aspelin , et al., “Kidney Disease: Improving Global Outcomes (KDIGO) Acute Kidney Injury Work Group. KDIGO Clinical Practice Guideline for Acute Kidney Injury,” Kidney International in Supplements (2011) 2, no. 1 (2012): 1–138, 10.1038/KISUP.2012.1.

[12] P. Osmancik , B. Bacova , M. Hozman , et al., “Myocardial Damage, Inflammation, Coagulation, and Platelet Activity During Catheter Ablation Using Radiofrequency and Pulsed‐Field Energy,” JACC: Clinical Electrophysiology 10, no. 3 (2024): 463–474, 10.1016/J.JACEP.2023.11.001/SUPPL_FILE/MMC1.DOCX. 38085214

[13] Y. Takahashi , A. Takahashi , T. Kuwahara , et al., “Renal Function After Catheter Ablation of Atrial Fibrillation,” Circulation 124, no. 22 (2011): 2380–2387, 10.1161/CIRCULATIONAHA.111.047266. 22042886

[14] I. D. Bucaloiu , H. L. Kirchner , E. R. Norfolk , J. E. Hartle , and R. M. Perkins , “Increased Risk of Death and De Novo Chronic Kidney Disease Following Reversible Acute Kidney Injury,” Kidney International 81, no. 5 (2012): 477–485, 10.1038/ki.2011.405. 22157656

[15] S. N. Heyman , S. Rosen , S. Fuchs , F. H. Epstein , and M. Brezis , “Myoglobinuric Acute Renal Failure in the Rat: A Role for Medullary Hypoperfusion, Hypoxia, and Tubular Obstruction,” Journal of the American Society of Nephrology 7, no. 7 (1996): 1066–1074, 10.1681/ASN.V771066. 8829123

[16] H. R. H. De Geus , J. Bakker , E. M. E. H. Lesaffre , and J. L. M. L. Le Noble , “Neutrophil Gelatinase‐Associated Lipocalin at ICU Admission Predicts for Acute Kidney Injury in Adult Patients,” American Journal of Respiratory and Critical Care Medicine 183, no. 7 (2011): 907–914, 10.1164/RCCM.200908-1214OC;SUBPAGE:STRING:FULL. 20935115

[17] C. R. Parikh , S. G. Coca , H. Thiessen‐Philbrook , et al., “Postoperative Biomarkers Predict Acute Kidney Injury and Poor Outcomes After Adult Cardiac Surgery,” Journal of the American Society of Nephrology 22, no. 9 (2011): 1748–1757, 10.1681/ASN.2010121302. 21836143 PMC3171945

[18] J. L. Koyner , V. S. Vaidya , M. R. Bennett , et al., “Urinary Biomarkers in the Clinical Prognosis and Early Detection of Acute Kidney Injury,” Clinical Journal of the American Society of Nephrology 5, no. 12 (2010): 2154–2165, 10.2215/CJN.00740110. 20798258 PMC2994075

[19] V. S. Sabbisetti , S. S. Waikar , D. J. Antoine , et al., “Blood Kidney Injury molecule‐1 Is a Biomarker of Acute and Chronic Kidney Injury and Predicts Progression to ESRD in Type I Diabetes,” Journal of the American Society of Nephrology 25, no. 10 (2014): 2177–2186, 10.1681/ASN.2013070758/-/DCSUPPLEMENTAL. 24904085 PMC4178434

[20] R. A. Zager , “Rhabdomyolysis and Myohemoglobinuric Acute Renal Failure,” Kidney International 49, no. 2 (1996): 314–326, 10.1038/ki.1996.48. 8821813

[21] T. Ninomiya , V. Perkovic , B. E. De Galan , et al., “Albuminuria and Kidney Function Independently Predict Cardiovascular and Renal Outcomes in Diabetes,” Journal of the American Society of Nephrology 20, no. 8 (2009): 1813–1821, 10.1681/ASN.2008121270. 19443635 PMC2723977

[22] S. Bolisetty and A. Agarwal , “Urine Albumin as a Biomarker in Acute Kidney Injury, American Journal of Physiology,” Renal Physiology 300, no. 3 (2011): 626–627, 10.1152/AJPRENAL.00004.2011. PMC3064133 21228105

[23] O. S. Better and Z. A. Abassi , “Early Fluid Resuscitation in Patients With Rhabdomyolysis,” Nature Reviews: Nephrology 7, no. 7 (2011): 416–422, 10.1038/nrneph.2011.56. 21587227

[24] I. Kawamura , S. Miyazaki , R. Kato , et al., “Comparison of Hemolysis With Different Pulsed Field Ablation Systems,” Heart Rhythm 23, no. 2 (June 2025): 388–395, 10.1016/J.HRTHM.2025.05.072. 40472952

[25] L. Marcon , D. G. Della Rocca , G. Vetta , et al., “Analysis of Hemolysis Following Pulse Field Ablation Using Three Different Technologies,” supplement, Europace 27, no. S1 (2025): euaf085.437, 10.1093/EUROPACE/EUAF085.437.

